# Ubiquitous Robotic Technology for Smart Manufacturing System

**DOI:** 10.1155/2016/6018686

**Published:** 2016-06-30

**Authors:** Wenshan Wang, Xiaoxiao Zhu, Liyu Wang, Qiang Qiu, Qixin Cao

**Affiliations:** Research Institute of Robotics, Shanghai Jiao Tong University, Shanghai 200240, China

## Abstract

As the manufacturing tasks become more individualized and more flexible, the machines in smart factory are required to do variable tasks collaboratively without reprogramming. This paper for the first time discusses the similarity between smart manufacturing systems and the ubiquitous robotic systems and makes an effort on deploying ubiquitous robotic technology to the smart factory. Specifically, a component based framework is proposed in order to enable the communication and cooperation of the heterogeneous robotic devices. Further, compared to the service robotic domain, the smart manufacturing systems are often in larger size. So a hierarchical planning method was implemented to improve the planning efficiency. A test bed of smart factory is developed. It demonstrates that the proposed framework is suitable for industrial domain, and the hierarchical planning method is able to solve large problems intractable with flat methods.

## 1. Introduction

As the ear of Industry 4.0 comes, industrial robots are no longer the preprogrammed robots setting separately doing their repeating jobs [1, 2]. As the manufacturing tasks become more individualized and more flexible, it shows great prospect to develop smart manufacturing systems, where machines are not likely to be preconfigured by traditional teaching methods, but doing variable tasks and coping with a wide variety of unexpected environmental and operational changes. The future manufacturing industry also requires that the system could dynamically schedule the tasks for these machines according to their work loads and the received tasks.

This feature of doing various tasks utilizing collaboration of distributed devices shares common ideas with the ubiquitous robotic technology, which is mainly applied in service robots domain [3]. In this perspective, the novel industrial manufacturing system could take advantages of the ubiquitous robotic technology.

In a typical ubiquitous robotic system, robotic devices are developed into modules [4–6]. These modules are connected through network, enabling data sharing and functionality calling. This modularized framework, which brings painless modification, expansion, and deletion, could also be applied to the smart manufacturing domain. We propose in this paper a framework of smart factory that takes advantage of a component based method, which abstracts each machinery process as a module with standardized communication ports. So different machines are able to communicate and cooperate with each other upon these ports.

Another important issue of ubiquitous robotic systems is the development of a task level learning and planning module that handles various tasks and dynamic environment without recoding the robots [7, 8]. This is also critical for smart factories, where there may be a variety of orders and different situations for each order. For example, in a future smartphone assembly factory, customers could make highly customized orders, such as individualized color, button shape, and cover material. The manufacturing process could be varied from order to order. In addition, it should take processing failures, human interferences, order changes, and other uncertainties into consideration. As a result, the task planning module for large-scale problems with uncertainty shows great importance.

Compared to the ubiquitous robotic systems, the task planning in industrial domain is even more challenging due to its larger planning space. For example, even in a small and medium factory, there could be dozens of machinery process and the planning space grows exponentially. Notice that human solves tasks in a hierarchical way, and fortunately most tasks in industrial domain have hierarchical structures. As a result, the large task can be divided into a task tree consisting of small subtasks, which can be solved more efficiently. Furthermore, some subtasks are reusable among up level tasks. In this study, a hierarchical task planning method is proposed to improve the planning efficiency. A study case of the smart assembly line is implemented as a demonstration platform for our methods.

## 2. Related Works

Many existing studies on smart factory focus on how to integrate RFID into the manufacturing system to collecting more data [9–11]. The manufacturing is smarter by tracking the processing information. We argue that it would achieve higher flexibility and intelligence if connecting not only the production but all the machinery processes. So different robotic devices could collaborate into different groups according to different tasks.

The ubiquitous robotic technology is widely studied these years. A number of frameworks have been proposed [12–14]. Recently, more efforts have been made on task level planning and learning technologies. The task planning methods for such multiagent systems could be categorized into centralized planning and decentralized planning. Decentralized planning methods are mainly applied to loosely coupled problems such as multi-UAV environmental monitoring [15] and cooperative mapping and localization [16]. As the individual machinery processes are highly coupled in manufacturing tasks, we prefer the centralized planning method.

The most commonly employed centralized techniques are based on automated planning in Artificial Intelligence. Ha et al. used SHOP2 planner to decompose services based on semantic knowledge ‎[12]. Erdem et al. presented an application of answer set programming to housekeeping robotics [17]. Niemueller et al. approached the task planning problem by deploying a rule engine ‎[18]. These planning methods cannot deal with dynamic situations with uncertainties as is the case in the real world. In response to this, some researchers have used probabilistic models in task planning problems. For example, Barbosa et al. used Partially Observable Markov Decision Processes (POMDP) to model the tasks with uncertainty ‎[19]. Cirillo et al. implemented RTL plan for probabilistic domains [20]. Planning methods based on probabilistic models such as Markov Decision Process (MDP) model and POMDP model can handle nondeterministic problems but at significant cost. They suffer dimension explosion, which limits the size of the state space to impractical applications.

The researches of MDP planning methods for large problems mainly consist of two kinds, the state approximation and hierarchical planning. The former has considerable difficulty in applying to general purpose planner discussed in this study. So we focus on the hierarchical planning methods.

The efforts of achieving the hierarchical planning of MDP problems are divided into two parts: first, how to automatically generate the hierarchical structures [21, 22]; second, how to develop planning algorithms to solve subproblems introduced by the hierarchical structure [23, 24]. Sutton et al. ‎[25] used options to temporally abstract knowledge based on Semi-Markov Decision Process (SMDP) theory. Parr ‎[26] developed an approach to hierarchically structuring MDP policies called Hierarchies of Abstract Machines. Dietterich ‎[23] developed another approach called the MAXQ Value Function Decomposition. These methods assume the hierarchy is predefined by human experts. For the automatic task decomposition problem, Hengst ‎[27] proposed the HEXQ approach for the construction of a hierarchy of abstractions based on the change frequency of state variables. Jonsson ‎[28] proposed the VISA approach for decomposing factored MDPs based on causal relations between variables. Kheradmandian and Rahmati ‎[21] incorporated and represented the ability of data mining techniques in automatic discovering of structures and patterns. Most of these methods are based on statistic methods that try to learn the critical states as the subgoals. This learning process is time consuming and does not have any optimality guarantees. We followed the research of Hengst and Jonsson, who generate the task hierarchy depending on state variables. We improve Jonsson's work by abstracting hierarchical options instead of searching for exits. Consequently the optimality is improved from recursively optimal to hierarchically optimal.

## 3. System Architecture

**Figure 1:**
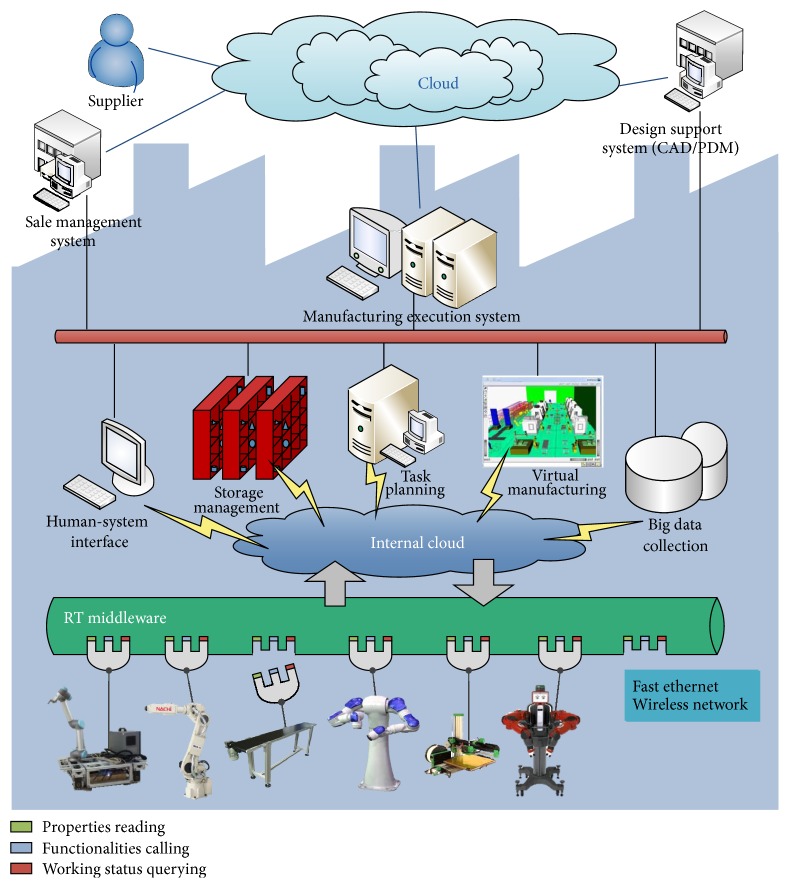
System architecture of the smart factory.

In contrast to traditional manufacturing processes, the smart manufacturing offers the advantage of distributed networked machines to complete different tasks through collaboration. The framework for smart factory is designed as in Figure 1.

In the low level, the robotic devices are developed into components that they can “plug and play” in the system and be reused and reconfigured according to different manufacturing process. These components are the foundation of the system. As mentioned, robotic components are highly heterogeneous with respect to platforms such as operating system, programming language, and communication media. Middleware is thus employed to generalize the components into a uniform abstraction which enables dynamic communication and coordination between any two of the modules ‎[29]. This also brings benefits to the modification of existing devices and the expansion of new ones.

In the middle level, a number of functionalities are developed in the internal cloud, such as the human-system interface, storage management, task planning, virtual manufacturing, and big data collection. The customer orders products through a human-system interface. The order includes customized requests, for instance, the favorite color and shape of the parts and whether the parts are being polished and so forth. These orders are sent to the task planning module, which also utilizing the information from the storage management module. The planner is the key part of the system's agility and intelligence. It turns customers' orders into subtask sequences, which can be directly carried out by corresponding robotic components. It is a general purpose planner based on Reduced Markov Decision Process (RMDP) model, which will be detailed later.

In the upper level, there are manufacturing execution system, sale management system, and design support system. These are all critical part of the industrial production process. This paper will not get into details of these big systems but mainly focus on the task planning module and the component based technology.

## 4. Component Based Machinery Process

Components use ports to communicate with each other and with high level controller. The ports are categorized into data ports and service ports ‎[30]. The data port is responsible for the continuous exchange of data. Each component can have any number of data in-ports and out-ports. A data out-port sends the data to a corresponding in-port which receives the data. The service port provides the command based communication. The component with a service port, offering a set of services, listens for requests for those services via a connector.

Each component has three service ports, namely, FuncGet, FuncSet, and ExeStatusGet. The service port is responsible for the interaction with the upper layer. FuncGet port reports to the service layer about the components' state. For example, the polishing robot reports the available polishing configuration; the Autonomous Intelligent Mobile Manipulator (AIMM) reports its states including its coordinates, whether the manipulator is empty, and battery level. FuncSet port provides the functionality invoking, such as setting the target position for the AIMM, starting polishing with certain configuration, and so forth. ExeStatusGet port returns the execution status, for example, whether or not the AIMM has reached its destination, or whether the polishing robot succeeds or fails in doing the task.

Each component may have any number of data ports for continuous data exchange between components. For instance, the localization information is transferred from the data out-port of laser component to the data in-port of the path planning component. Once two data ports are connected, those two components are able to perform real-time communication to accomplish the task collaboratively.

**Figure 2:**
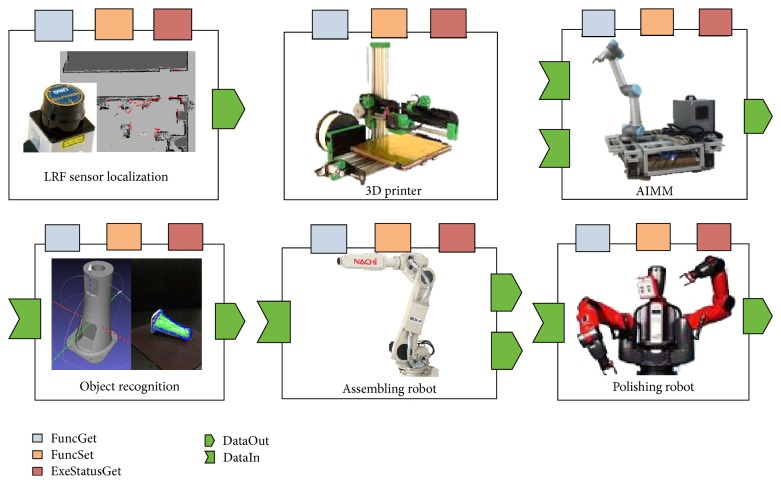
Robotic components in our system.

The individual robotic functions are also critical to the system's intelligence. The traditional industrial robots are like blind and deaf muscles repeating some predefined motions. In the smart factory, robotic components are capable of sensing the environment and making decisions in optimization of resources and time. Some of the robotic components in our system are shown in Figure 2.

There are five 3D printers with materials in different colors, one dual-arm robot for polishing, one assembling robot, and one AIMM. The AIMM is equipped with laser sensor for localization and obstacles avoiding. Other software components such as localization, path planning, and object recognition are also implemented.

### 4.1. Polishing Component with Auto Path Generation

**Figure 3:**
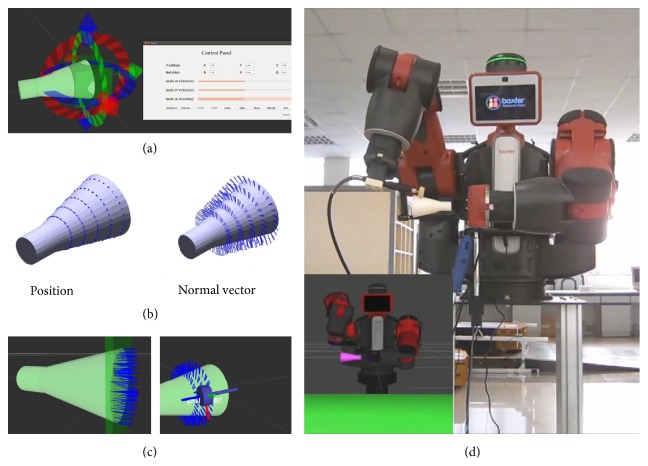
(a) Configuring the polishing area, (b) auto-generating the polishing path, (c) path generation and tool simulation, and (d) motion planning and polishing with dual-arm robot.

Traditionally, the polishing path is taught by the expert engineers. This teaching process could be complex and tedious. In our smart factory, the polishing path is automatically generated from the CAD data (Figures 3(b) and 3(c)). Then, the robot follows this path by a motion planning algorithm with collision avoidance (Figure 3(d)). Besides, the polishing area is easy to specify with a user-friendly GUI as in Figure 3(a).

### 4.2. AIMM Component

**Figure 4:**
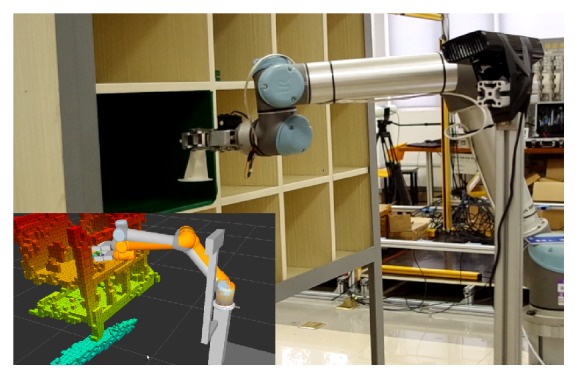
AIMM is picking up a working part form the warehouse.

AIMM is responsible for the transportation task that transports parts and work pieces between workstations and storages (Figure 4). Such transportation tasks contain physical separation larger than the workspace of the robot manipulator. This requires a lot of technologies such as object recognition, grasp point generating, motion planning, localization, and path planning. It uses RGB-D camera to do the object recognition and obstacle avoidance and uses laser sensor to do the localization.

### 4.3. Assembling Component

**Figure 5:**
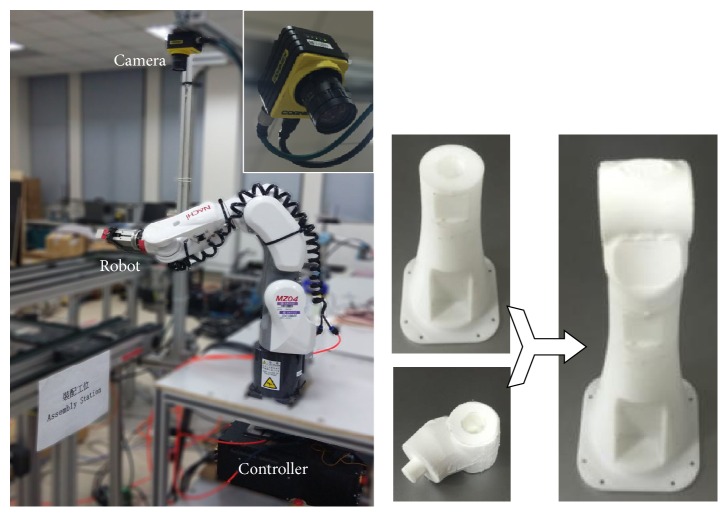
Assembling robot with visual detection.

The assembling robot also has the sensing capbility (Figure 5). It grasps the working parts by online detecting the location and orientation. The visual detection is based on template matching method and is able to recognize complex shape with localization error below 1 mm. We also employ a motion planning and motion controlling module for assembling and obstacle avoiding.

## 5. Hierarchical Task Planning

The challenges of task planning for smart factory domains are introduced by their large problem size and uncertainty. This study follows the techniques of automated planning derived from the AI field. Firstly, a task model called RMDP model is proposed. This model is designed for describing problems with large size and limited uncertainties such as smart factory. Secondly, the relations of variables are analyzed based on this model. The relations are depicted by the causal graph. Thirdly, according to the causal graph, the original actions are hierarchically abstracted into options, which induce smaller subproblems. At last, the subproblems and the original problem are solved based on Semi-MDP theory.

### 5.1. Task Modeling Based on Multivalued State Variables

The task planning problem is modeled as a state transition system. Depending on different assumptions, various models are proposed. The two most commonly used models are the classical planning model and the MDP model. However, the classic model cannot deal with dynamic situations with uncertainties as is the case in the real world. The MDP model supports nondeterministic actions and dynamic situations, but it scales poorly to large problems.

We propose the RMDP model by making the following assumption in line with the manufacturing domains. It is assumed that, after actions are executed by robotic components, the outcome could be among a few predictable states, which are the successful state and a few failed states. This assumption simplifies the MDP model by decreasing the branching factor of the state space. In addition, the model is designed based on multivalued state variables, which is more compact and natural compared to the propositional based models. This is important for the following variable analysis.

**Definition 1 .**

*RMDP model* is defined as a five-tuple Π = (*V*, *D*, *A*, *I*, *G*):

(i) *V* = {*v*_1_, *v*_2_,…, *v*_*n*_} is a finite set of state variables;
(ii) *D* = {*d*_1_, *d*_2_,…, *d*_*n*_} is a finite set of variable domains, each *v*_*i*_ ∈ *V* with a finite domain *d*_*i*_ ∈ *D*. *V* and *D* define the planning space *S*, where state *s* ∈ *S* is represented as a vector [*x*_1_, *x*_2_,…, *x*_*n*_], where *x*_*i*_ ∈ *d*_*i*_ is the value of variable *v*_*i*_;
(iii) *A* = {*a*_1_, *a*_2_,…, *a*_*m*_} is a finite set of actions; each *a*_*i*_ ∈ *A* is a triple (pc, ef, *c*) referring to the action's preconditions, effects, and cost, respectively. The preconditions of action *a* are defined with a list [pc_1_, pc_2_,…, pc_*j*_], where pc_*i*_ = (*v*, *x*) denotes that the value of variable *v* should be *x* to satisfy the precondition. The effects of action *a* are defined with an effect list [*e*_1_, *e*_2_,…, *e*_*k*_], where *e*_*i*_ = (*p*, *v*, *x*) denotes that the variable *v* will change its value to *x* with probability *p*, after the action's execution. Each action has a cost *c*, which acts like a reward function in MDP model;
(iv) *I* ∈ *S* denotes the initial state;
(v) *G*⊆*S* denotes the set of goal states.

**Figure 6:**
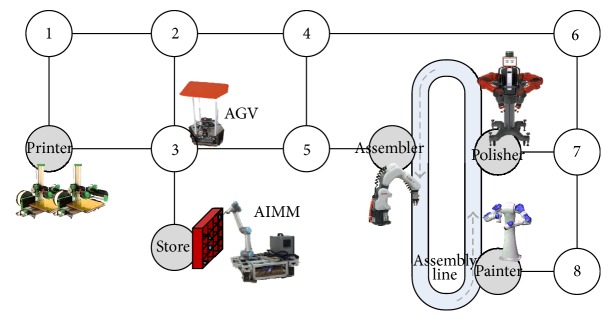
The smart factory planning domain.

The demonstrating task in this study is shown in Figure 6. As described in Section 4, the smart factory in our study case includes 3D printers, polishing robot, assembling robot, and AIMM. This task is designed according to the physical system in our laboratory, which will be detailed in the next section. One workstation of painting robot and one Automatic Guided Vehicle (AGV) are added to increase the complexity. Detailed task description is shown in Tables 1 and 2.

These actions in Table 2 are grounding actions. There are too many grounding actions to be defined by hand. In practice, the actions are defined in lifted manner. For example, there are 28 “Move” actions in total, 14 for AIMM and 14 for AGV. These 28 actions are presented by one lifted “Move” action: Move(Robot, Location, Location), where the action is parameterized with variable types “Robot” and “Location”. The lifted actions are compiled to the grounding actions in a preprocessing stage.

### 5.2. Variable Dependency Analysis

Notice that there are dependencies between different variables. For example, according to the “pickup” action, the change of value of “part1_loc” is dependent on the value of “AIMM_loc”. According to “polish” action, the change of value of “polished_part1” is dependent on the value of “part1_loc”. We depict these dependencies by a causal graph, following the work of Helmert ‎[31] and Jonsson ‎[28].

**Definition 2 .**

The* causal graph* of ∏ is a directed graph CG(∏) with vertices *V*_cg_ and an arc(*u*, *v*) whenever there exists an action *a* ∈ *A* so that either (i) there exists *a* ∈ *A* so that *u* ∈ *a*(pc) and *v* ∈ *a*(ef) are both defined, or (ii) there exists *a* ∈ *A* so that *u* ∈ *a*(ef) and *v* ∈ *a*(ef) are both defined.

**Figure 7:**
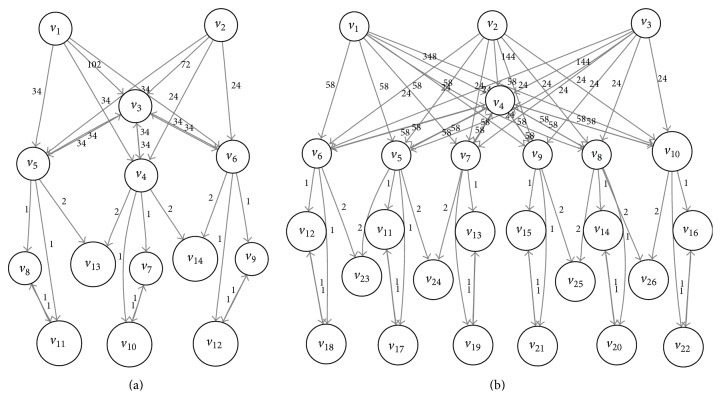
Causal graphs of smart factory tasks.

The causal graph is independent of the initial state and goal state. As a result, it can be calculated offline. The causal graph of the example task is shown in Figure 7(a), where each circle represents a variable associated with Table 1. If we add one more AGV and three more parts to the factory (the variables are listed in Table 3) and the causal graph is shown in Figure 7(b), the associated algorithm is as Algorithm 1 shows.

The causal graph reflects the structure of the planning problem. The overall task is decomposed according to the causal relations of variables.

**Figure 8:**
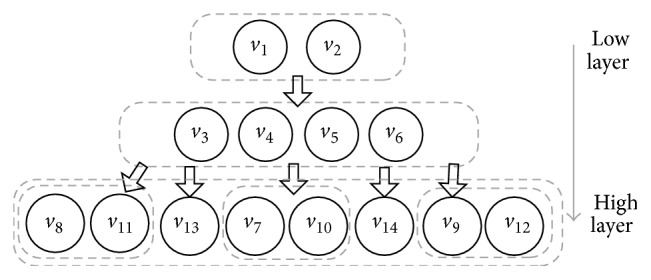
Combine the variables in strongly connected components.

If the causal graph is acyclic, the decomposition is very intuitive. The task can be decomposed into the same structure as the causal graph. Then the task could be solved hierarchically. However, most tasks have a cyclic causal graph, such as in Figure 7. In these cases, we find out all the strongly connected components (SCCs) in the causal graph and combine the variables in each SCC. As a result, the task of Figure 7(a) is decomposed as in Figure 8. Because the *v*_7_–*v*_14_ in the high layer all have very small domain size, we combine them in one layer. Because these SCCs do not have interdependencies, the combination will not change the number of subtasks, but only for structural simplicity.

Given the task structure, the task is able to be solved hierarchically from low layer to the high layer. This process is divided into two phases called iteratively. These two phases are abstracting options and solving Semi-MDP, which will be detailed in the following two sections.

### 5.3. Hierarchical Option Causal Abstraction

Given the hierarchical structure, one of the key problems is to find the reusable subtasks. So the original problem could be decomposed into the combination of these subtasks. We propose an algorithm called Hierarchical Option Causal Abstraction (HOCA). In general, the actions are abstracted into hierarchical options based on causal relations. Each option induces a subtask that could be solved offline. The planning efficiency is remarkably improved using these options instead of the primitive actions. Options are used for the generalization of temporally extended primitive actions by Sutton et al. ‎[25]. In their work, options are designed by human expert. This term is modified in this study in order to enable the automatic abstraction.

**Definition 3 .**

An* option* is a four-tuple *o* = (fa, pc, *β*, *π*), where

(i) fa denotes the father option of *o*;
(ii) pc is the preconditions of option *o*, similar with the action's definition;
(iii) *β* is the set of goals of option *o*, each “var-value” pair (*v*, *x*) ∈ *β* requiring that the goal value of variable *v* is *x*;
(iv) *π* : *s* ↦ *o* is the policy for this option, which is calculated by the method detailed in the next section.

**Definition 4 .**

The* option hierarchy* derived from action *a* is represented as *H* ^*a*^ = {*o*_0_^*a*^, *o*_1_^*a*^,…, *o*_*m*_^*a*^}, where *o*_0_^*a*^ = ([ ], *a*(pc), *a*(ef), [ ]) is directly converted from action *a*, and ∀1 < *k* ≤ *m*, there is *o*_*k*_^*a*^(fa) = *o*_*k*−1_^*a*^.

To automatically generate options, we define the preconditions of the action as a subgoal based on the causal relations of the variables. This subgoal is solved within an abstracted state space, which is much smaller than the original space. Through this process, a primitive action is abstracted into an option, which could be further abstracted into higher level options. These options derived from action *a* form an option hierarchy as Definition 4.

Practically, in each layer *k*, we further define the abstract option (ABO) and active option (ACO). Each ABO derives a hierarchical option in layer *k*, while ACO is used for solving the Semi-MDP, which will be detailed in the next section. Assume the hierarchical structure is *Ζ* = {*V* ^0^, *V* ^1^,…, *V* ^*L*^}; *V* ^*k*^ represents the set of variables in the *k*th layer. Define *V* ^*k*−^ = ⋃_*i*=1_^*k*^ *V* ^*i*^ as the union of the variables in layer lower than or equal to *k*. Define *V* ^*k*+^ = ⋃_*i*=*k*+1_^*L*^ *V* ^*i*^ as the union of the variables in layer higher than *k*. Define *V* ^*a*^ as the set of variables appearing in action *a*'s preconditions and effects.

**Definition 5 .**

The hierarchical option *o* ^*a*^ at layer *k* is an* active option* (ACO) if and only if *V* ^*a*^⊆*V* ^*k*−^. The set of ACOs in layer *k* is denoted by ${\hat{O}}^{k}$.

**Definition 6 .**

The hierarchical option *o* ^*a*^ at layer *k* is an* abstract option* (ABO) if and only if *o* ^*a*^(pc)∩*V* ^*k*^ ≠ *⌀* and *V* ^*a*^∩*V* ^*k*+^ ≠ *⌀*. The set of ABOs in layer *k* is denoted by ${\tilde{O}}^{k}$.

In layer *k*, the ACOs are options that all the associated variables are within the *k*th and above layer. So the ACO in layer *k* is fully abstracted. It can be used for solving the Semi-MDPs. The ABOs of layer *k* are options that satisfy two conditions: firstly existing variable both in the option's precondition and in layer *k*; secondly existing variable in higher layer than *k*. Following the definitions, the hierarchical option in the *k*th layer is derived as Algorithm 2.

For example, in the low layer of the smart factory task, all the “move” actions are ACOs. The “pickup” and “putdown” actions satisfy the ABO conditions. The action “Pickup_AIMM_store_part1” is abstracted to option, which induces “AIMM_loc = store_spot” as a subgoal. Similarly in layer two, all the “pickup” and “putdown” options become ACOs. The actions “polish”, “paint”, and “assemble” are ABOs abstracted into options.

The options of one task are also independent of the initial and goal states, but they only depend on the task definitions of variable, domain, and actions. As a result, they can be calculated offline.

### 5.4. Solve the Hierarchical Semi-MDPs

A Semi-Markov Decision Process (SMDP) is a MDP model with temporally extended actions ‎[32]. Efforts have been done to extend planning algorithms from MDP to SMDP problems ‎[25]. As described above, the options abstracted in this study are also temporally extended. The subtasks relying on these options are consequently SMDPs.

**Definition 7 .**

The* SMDP* problem is defined as four-tuple Σ = (*V*, *D*, *O*, *β*) where *V* is the variable set; *D* is the domain set; *O* is the option set; *β* is the goal for this problem.

The solution of a SMDP problem is a policy *π*, mapping from states to options. To calculate the policy there are a bunch of algorithms extended from MDP problems, such as Dynamic Programming based on Bellman equation. For any state *s* ∈ *S*, the value function of policy *π* is

(1) VFπs=Ert+rt+1+⋯+rt+k+VFπst+k ∣ επs,s,t=cπss+∑s′∈Spπss′ ∣ sVFπs′,

where *ε*(*π*(*s*), *s*, *t*) denotes the event of executing *π*(*s*) in state *s* at time *t* and *t* + *k* is the random time at which *π*(*s*) terminates. *c* ^*o*^(*s*) and *p* ^*o*^(*s*′∣*s*) denote the cost and transition probability of option *o*. They compose the option's model.

The optimal value function is the one with maximum value

(2) VF∗s=maxπ ⁡VFπs=maxπ ⁡Ert+rt+1+⋯+rt+k+VFπst+k ∣ επs,s,t=maxπ⁡cπss+∑s′∈Spπss′ ∣ sVFπs′.

The optimal policy is the one that maximizes the value function

(3) π∗arg⁡maxπ ⁡VFπs=arg⁡maxπ⁡cπss+∑s′∈Spπss′ ∣ sVFπs′.

To calculate the policy, one important issue is how to get the models of the options in the option set. Since option *o* is abstracted from a hierarchy, the outcome state *s*′ and the option cost *c* ^*o*^(*s*) are random variables. According to Sutton et al. ‎[25], they proposed a multitime model

(4) $$\begin{array}{c} p^{o}\left(\;s^{\prime }\;|\;s\;\right)=\overset{\infty }{\underset{k=1}{\sum }}p\left(\;s^{\prime },k\;\right)\gamma^{k}, \end{array}$$

where *p*(*s*′, *k*) is the probability that the option terminates in *s*′ after *k* steps and *γ* is a discount factor. On the other hand, the cost of *o* is a function of the state *s*:

(5) $$\begin{array}{c} c^{o}\left(\;s\;\right)=E\left\{\;c_{t+1}+c_{t+2}+\cdots +c_{t+k}\;|\;\varepsilon \left(\;o,s,t\;\right)\;\right\}, \end{array}$$

where *ε*(*o*, *s*, *t*) denotes the event of *o* being executed in state *s* at time *t* and *t* + *k* is the random time at which *o* terminates.

The subtask induced by abstract option *o* in layer *k* is denoted by $\Sigma_{o}^{k}=(V^{k-},D^{k-},{\hat{O}}^{k},o(\beta ))$, where ${\hat{O}}^{k}$ is the ACO set in this layer and *o*(*β*) is the subgoal of option *o*. The variables and domains are all subset of the original problem. This makes the state space smaller.

**Figure 9:**
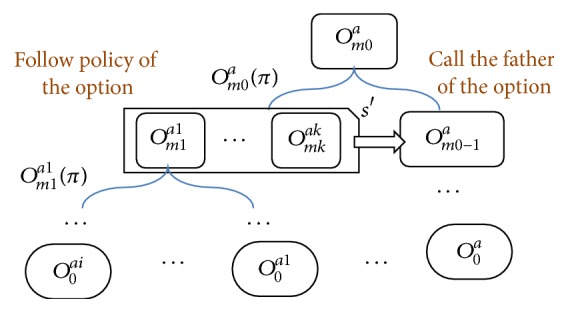
Execution of option *o* forms an execution tree.

According to the option hierarchy in Definition 4, the execution of *o* forms an execution tree. It is a recursive process as Figure 9 shows. The execution consists of two phases. It firstly follows the policy of the option and secondly calls the father option. This process is done recursively until all options reach down to the primitive actions, which lie on the leaf nodes of the execution tree. For the leaf nodes, the costs are equal to the cost of the primitive actions. In practice, the trees usually are not very deep, two or three layers in our example.

As a result, let *o*′ be the father of *o*; the cost of option *o* is represented by the following recursive formula:

(6) cosVFoπs+Eco′πs′=VFoπs+∑s′po′πs′ ∣ sco′πs′,

where *s*′ is a random variable that denotes the state at which it terminates when satisfying *o*(*β*); and *p* ^*o*′(*π*)^(*s*′∣*s*) is probability that the state terminates at *s*′ following the policy *o*′(*π*).

However in practice, the above model is difficult to compute. One solution is to employ model-free algorithms such as Temporal Difference (TD) and *Q*-learning. Instead of calculating, it just observes the outcome state and cost and updates the value function with small steps. The TD form of the updating rule is

(7) $$\begin{array}{c} {\text{VF}}^{\pi }\left(\;s\;\right)=\left(\;1-\alpha \;\right){\text{VF}}^{\pi }\left(\;s\;\right)+\alpha \left[\;c^{\pi \left(s\right)}\left(\;s\;\right)+{\text{VF}}^{\pi }\left(\;s^{\prime }\;\right)\;\right], \end{array}$$

where

(8) $$\begin{array}{c} c^{o}\left(\;s\;\right)={\text{VF}}^{o\left(\pi \right)}\left(\;s\;\right)+c^{o^{\prime }\left(\pi \right)}\left(\;s^{\prime \prime }\;\right), \end{array}$$

where *α* is the step size, *o*′ = *o*(fa), *s*′ is the outcome state after the hierarchical tree of *o* has completed, and *s*′′ is the outcome state after policy *o*(*π*) has terminated.

Similarly, the *Q*-learning version of the updating rule is

(9) $$\begin{array}{c} {\text{VF}}^{\pi }\left(\;s\;\right)=\left(\;1-\alpha \;\right){\text{VF}}^{\pi }\left(\;s\;\right)+\alpha \;\underset{o}{\max}\left[\;c^{o}\left(\;s\;\right)+VF^{\pi }\left(\;s^{\prime }\;\right)\;\right]. \end{array}$$

The *Q*-learning algorithm for SMDP problem is as in Algorithm 3.

After the SMDP for option *o* has been calculated, this option has been abstracted in the current layer. It may become an ACO of the higher layer or be abstracted again in higher layer. In summary, this abstracting process and calculating SMDP process are called iteratively from low layer to high layer as Algorithm 4 shows.

Although the planning efficiency is remarkably improved, the policy achieved by HOCA algorithm is not a global optimal policy. As many hierarchical planners do [26, 27], HOCA achieves a hierarchical optimal policy. This means that the solution is optimal given the constraints of the hierarchy. It is often a tradeoff. If we want a policy that is closer to the global optimal one, we should use simpler hierarchy with options of lower abstraction level. But if the speed is more wanted, it needs more abstract options.

## 6. Experiments and Results

**Figure 10:**
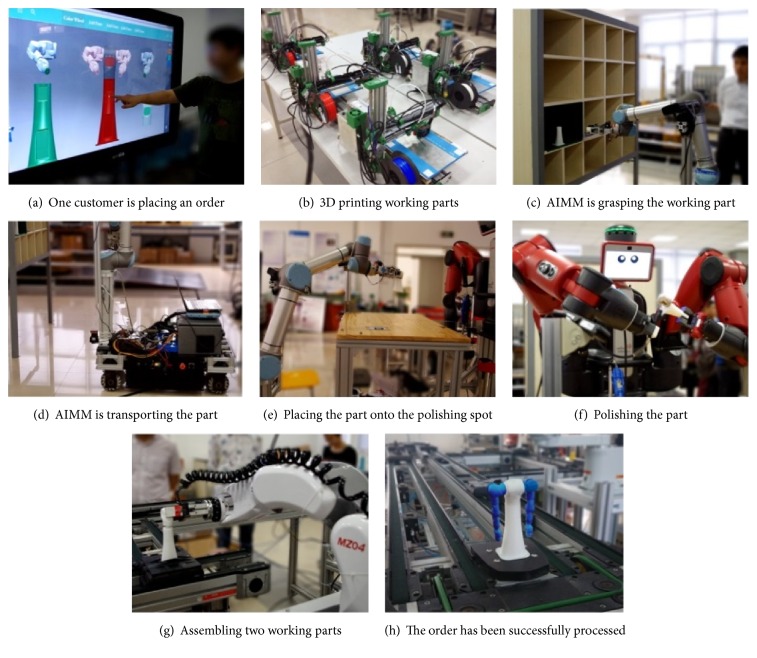
Manufacturing process in smart factory.

A smart factory was implemented based on the ubiquitous robotic technology. A demonstration video could be found in the Supplementary Material available online at http://dx.doi.org/10.1155/2016/6018686 as well as in this link: https://youtu.be/MVO4yGF0GwY. It took in customers' individualized order and arranged the producing process accordingly.  Figure 10 shows one execution of the smart factory task. First, the customer made an order through the user interface. The order was then sent to the task planning module, which calculated the action sequence hierarchically. 3D printers started to make parts with specific color and shape as Figure 10(b). Meanwhile, the AIMM transported the part from the storage to the polishing station as shown in Figures 10(c)–10(e). After that, the dual-arm polishing robot polished the part according to customer's configuration as in Figure 10(f). At last, the parts were transported to the assembling spot after which the product was successfully processed as in Figures 10(g) and 10(h).

With the component based framework, every two of the robotic devices are ready to cooperate with each other. For instance, the continuous localization data is transferred from the laser sensor to the AIMM's path planning module through data port. And the AIMM can pass the working part directly onto the polishing robot, after calling on its service port. Further, this modular framework also facilitates the easy expansion of new devices and painless modification of the existing devices.

**Figure 11:**
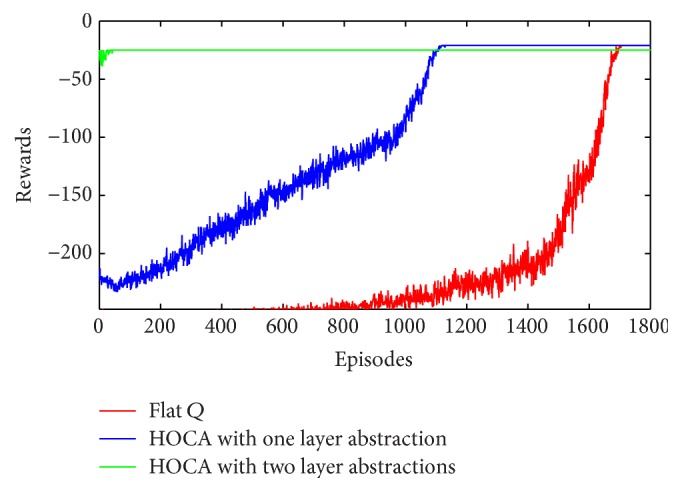
Performance of HOCA under different abstractions against a flat *Q*-learning.

The hierarchical task planning method decomposes the original big problem into a hierarchy of small problems. The problem of smart factory in Figure 6 has 2.4*e* + 8 states. It takes about 1700 episodes for the flat *Q*-learning algorithm to converge to the optimal value. HOCA algorithm is firstly run offline to compute the causal graph and hierarchical structure. Then according to the three-layer structure as Figure 8 shows, two layers of option abstraction are applied. Using HOCA algorithm described previously with one layer of abstraction, namely, just using the abstracted options “pickup” and “putdown”, it convergences in less than 1200 episodes. In this problem, it still converges to the optimal value using one layer of abstraction (Figure 11). When HOCA is run with two layers abstraction, in which case all the primitive actions including “polish”, “paint”, and “assemble” are abstracted, the convergence time is significantly reduced. This is because the domain sizes of the high layer variables are relatively small in this example. Using those abstracted options, it only takes 4 or 5 steps to reach the goal (Figure 11). However, it does not reach global optimal policy using two layer abstractions. In the optimal solution, three working parts are picked and placed on the AGV in sequence and transferred together. This strategy is unavailable when using highly abstracted options, in which the parts are transferred one by one. We plan to study this problem in the next step. It may achieve the global optimum if we flat the policy and refine it with low layer options.

The improvements are even larger on more complex tasks. In the smart factory task in Figure 7(b), the size of the state space is about 4.8*e* + 15. It is generally unsolvable for flat algorithms. But for HOCA, it will not be much harder than the previous task. The additional AGV and three working parts in this task have same domain and subtasks with the ones in the previous task. Those subtasks are reused and bring no extra computational loads for offline option abstractions. The online iteration is still sustainable.

## 7. Conclusions

Given the increasing popularity of smart manufacturing as a solution offering better autonomy, this paper discussed the similarity of the smart manufacturing with the ubiquitous robotic system. A component based framework has been proposed and proved to be applicable for industrial domain. Further, since the manufacturing problems are often in large size with uncertainties, a hierarchical task planning method called HOCA based on RMDP model has been developed. This method decomposes the original big problem into a hierarchy of small problems by automatically abstracting primitive actions to a hierarchy of options according to variable dependencies.

A smart factory was implemented as the testing bed of our framework and algorithms. The individualized orders were processed by the system that arranged the producing process accordingly. The results showed that the framework facilitates the communication and cooperation between the robotic components. Further the hierarchical planning method has remarkably reduced the problem size and makes large problem tractable. However, the planner can only obtain hierarchical optimal results. This is the obvious defects and should be improved in the future.

It is our view that the results obtained from this work represent a substantial improvement. This method is not restricted to the particular domain discussed in this paper. As such, these results could also be beneficial to the researchers attempting to design smart manufacturing systems for other complex tasks in large-scale environment.

## Supplementary Material

The video in supplementary material demonstrates a smart assembly line based on the ubiquitous robotic technology. Firstly, the customer made an order through the user interface. The order was then sent to the task planning module, which calculated the action sequence hierarchically. Then, the planning results were sent to the robotic components, such as the AIMM component, the polishing component and the assembling component. These components complete the task by cooperating with each other according to the task planning results.

## Figures and Tables

**Algorithm 1:**
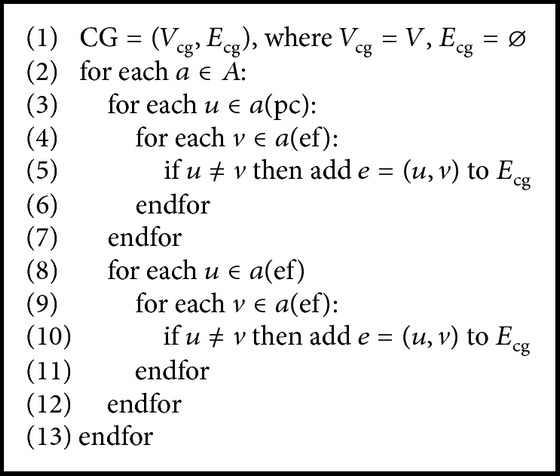
Calculate causal graph.

**Algorithm 2:**
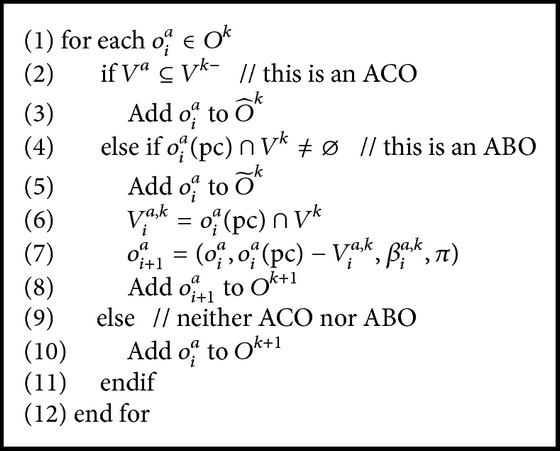
Abstract options in the *k*th layer.

**Algorithm 3:**
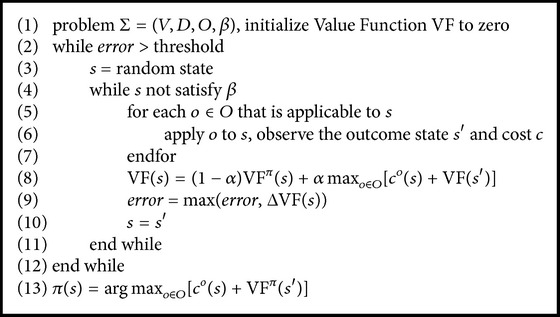
*Q*-learning for SMDP.

**Algorithm 4:**
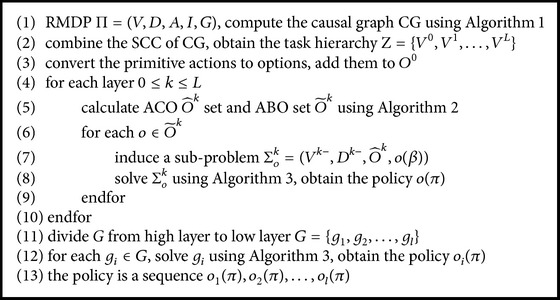
Hierarchical option causal abstraction.

**Table 1:** Variable *V* and domain *D* in the smart factory task.

	Variable	Domain
1	AIMM_loc	loc1, loc2,…, loc8, printer_spot, store_spot,…, painter_spot

2	AGV_loc	loc1, loc2,…, loc8, printer_spot, store_spot,…,painter_spot

3	AIMM_hand_empty	true, false

4	part1_loc	AIMM, AGV, printer_spot, store_spot, assembler_spot, polisher_spot, painter_spot
5	part2_loc	
6	part3_loc	

7	part1_color	red, blue, black, white
8	part2_color	
9	part3_color	

10	part1_polished	true, false
11	part2_polished	
12	part3_polished	

13	assembled_p1_p2	true, false

14	assembled_p1_p3	true, false

**Table 2:** Some of the actions in the smart factory task.

Action	Precondition	Effect	Probability
Move_AIMM_loc1_loc2	AIMM_loc = loc1	{AIMM_loc = loc2}	0.85
		{ }	0.1
		{AIMM_loc = printer_spot}	0.05

Move_AGV_loc3_loc5	AIMM_loc = loc3	{AIMM_loc = loc5}	0.85
		{ }	0.05
		{AIMM_loc = loc2}	0.05
		{AIMM_loc = store_spot}	0.05

Pickup_AIMM_store_part1	AIMM_loc = store_spot part1_loc = store_spot AIMM_hand_empty = true	{part1_loc = AIMM, AIMM_hand_empty = false} { }	0.9 0.1

Putdown_AIMM_painter_part3	AIMM_loc = painter _spot part3_loc = AIMM AIMM_hand_empty = false	{part3_loc = painter _spot, AIMM_hand_empty = true}	1.0

Putdown_AIMM_printer_part2_AGV	AIMM_loc = printer_spot AGV_loc = printer_spot part2_loc = AIMM AIMM_hand_empty = false	{part2_loc = AGV, AIMM_hand_empty = true} {part2_loc = printer_spot, AIMM_hand_empty = true}	0.9 0.1

Polish_part1	part1_loc = polish_spot part1_polished = false	{part1_polished = true, part1_color = white} { }	0.8 0.2

Paint_part1_red	part1_loc = paint_spot	{part1_color = red} { }	0.9 0.1

Assemble_p1_p2	part1_loc = assemble_spot part2_loc = assemble_spot assembled_p1_p2 = false	{assembled_p1_p3 = true} { }	0.95 0.05

**Table 3:** Variables of the smart factory task in Figure 7(b).

Tag	Variable
*v*_1_	AIMM_loc
*v*_2_	AGV1_loc
*v*_3_	AGV2_loc
*v*_4_	AIMM_hand_empty
*v*_5_–*v*_10_	part1_loc - part6_loc
*v*_11_–*v*_16_	part1_color - part6_color
*v*_17_–*v*_22_	part1_polished - part6_polished
*v*_23_	assembled_p1_p2
*v*_24_	assembled_p1_p3
*v*_25_	assembled_p4_p5
*v*_26_	assembled_p4_p6
